# Development of a Deep-Sea Submersible Chemiluminescent Analyzer for Sensing Short-Lived Reactive Chemicals

**DOI:** 10.3390/s22051709

**Published:** 2022-02-22

**Authors:** Lina Taenzer, Kalina Grabb, Jason Kapit, William Pardis, Scott D. Wankel, Colleen M. Hansel

**Affiliations:** 1 Woods Hole Oceanographic Institution, Marine Chemistry and Geochemistry, Woods Hole, MA 02543, USA; ltaenzer@whoi.edu (L.T.); kgrabb@whoi.edu (K.G.); sdwankel@whoi.edu (S.D.W.); 2 Massachusetts Institute of Technology, Earth, Atmospheric and Planetary Sciences, Cambridge, MA 02139, USA; 3 Woods Hole Oceanographic Institution, Applied Ocean Physics and Engineering, Woods Hole, MA 02543, USA; jkapit@whoi.edu (J.K.); wpardis@whoi.edu (W.P.)

**Keywords:** superoxide, chemiluminescence, deep-sea

## Abstract

Based on knowledge of their production pathways, and limited discrete observations, a variety of short-lived chemical species are inferred to play active roles in chemical cycling in the sea. In some cases, these species may exert a disproportionate impact on marine biogeochemical cycles, affecting the redox state of metal and carbon, and influencing the interaction between organisms and their environment. One such short-lived chemical is superoxide, a reactive oxygen species (ROS), which undergoes a wide range of environmentally important reactions. Yet, due to its fleeting existence which precludes traditional shipboard analyses, superoxide concentrations have never been characterized in the deep sea. To this end, we have developed a submersible oceanic chemiluminescent analyzer of reactive intermediate species (SOLARIS) to enable continuous measurements of superoxide at depth. Fluidic pumps on SOLARIS combine seawater for analysis with reagents in a spiral mixing cell, initiating a chemiluminescent reaction that is monitored by a photomultiplier tube. The superoxide in seawater is then related to the quantity of light produced. Initial field deployments of SOLARIS have revealed high-resolution trends in superoxide throughout the water column. SOLARIS presents the opportunity to constrain the distributions of superoxide, and any number of chemiluminescent species in previously unexplored environments.

## 1. Introduction

One of the greatest advantages of in situ chemical sensors is their ability to enable observations under ambient conditions that could otherwise not be made [1,2,3,4,5]. Many traditional measurement methods in observational oceanography require the retrieval of water samples for later analyses. For some measurements this approach may skew the representation of natural conditions, and for others, such as the measurement of short-lived chemical species, is impractical or entirely impossible. The development of in situ sensors allows us to discern nuanced features of an environment in a nearly unaltered state, and provides an opportunity to gain a more integrated understanding of marine biogeochemistry. For instance, recent advances in in situ instruments have afforded new and high temporal resolution glimpses into the dynamics of chemical compounds, such as nitrate [5], carbonate, and sulfate [6], among others [7,8].

Superoxide ($O_{2}^{\cdot -}$) is a reactive oxygen species (ROS) which, on short temporal and spatial scales, exerts an important role in controlling the redox conditions of its surroundings [9,10]. In the marine environment, monovalent reduction reactions yielding measurable $O_{2}^{\cdot -}$ in seawater occur through a plethora of pathways including photoreactions with chromophoric dissolved organic matter, abiotic reactions involving reduced metals and sulfide, and numerous extracellular production pathways tied to the activity of various phytoplankton, bacteria, and shallow-water coral species, among others [11,12].

The unique roles and fates of $O_{2}^{\cdot -}$ are the result of its enhanced chemical reactivity in comparison to molecular oxygen. As a consequence of this reactivity, superoxide is an important redox mediator involved in either the oxidation or reduction of numerous elements (e.g., iron, manganese, carbon). Further, superoxide is an enigmatic compound as it appears to be essential to the normal physiological function of aerobic cells, notably in cell signaling processes, but simultaneously has the potential to do damage to biomolecules at elevated concentrations [13]. Antioxidants such as superoxide dismutase (SOD), which catalyzes the degradation of $O_{2}^{\cdot -}$ to oxygen and hydrogen peroxide, are thought to be primarily responsible for directly regulating levels within biological systems [14]. Previous research suggests that the ability to produce and enzymatically eliminate ROS was likely a trait that emerged in the earliest life forms, and contributed to shaping the evolution of oxygenic pathways on earth [15].

Although $O_{2}^{\cdot -}$ is an important and possibly ubiquitous compound in certain marine environments, exploration of its distribution in the sea has been limited by challenges posed by its short half-life [16,17]. Its rapid decay precludes the ability to collect waters from the environment for lab or shipboard analyses, but rather necessitates a method for in situ measurement near the source of production. Recently, open questions regarding the extent to which $O_{2}^{\cdot -}$ reflects and/or controls the health of marine organisms, and regulates the bioavailability of essential trace nutrients such as Fe, has driven the research and development of new approaches and sensors with which to make direct measurements of steady-state concentrations [10]. Recently, development of a submersible $O_{2}^{\cdot -}$ sensor enabled the first in situ measurements of $O_{2}^{\cdot -}$, revealing elevated steady-state concentrations in the surface oceans, as well as localized production associated with some coral species [18].

The ubiquity of $O_{2}^{\cdot -}$ in near-surface environments revealed through in situ sensing fits a growing perception of ROS as an influential compound in shaping the interaction between life and its surroundings. While the development of shallow instrumentation to analyze $O_{2}^{\cdot -}$ in shallow water environments has already greatly expanded our knowledge of its functions and dynamics, deciphering the roles of $O_{2}^{\cdot -}$ in organismal physiology and health and the biogeochemistry of the ocean more broadly, will require examination of environments beyond the shallow sunlit surface ocean. To this end, here we present the development of a new submersible oceanic chemiluminescent analyzer of reactive intermediate species (SOLARIS), capable of measuring $O_{2}^{\cdot -}$ and other analytes amenable to chemiluminescent detection to depths of up to 5900 m. SOLARIS advances current in situ instrumentation with the capability for in situ calibrations, and a maneuverable sampling wand which allows for high spatial resolution.

## 2. Materials and Methods

### 2.1. Design and Configuration of SOLARIS

**Figure 1:**
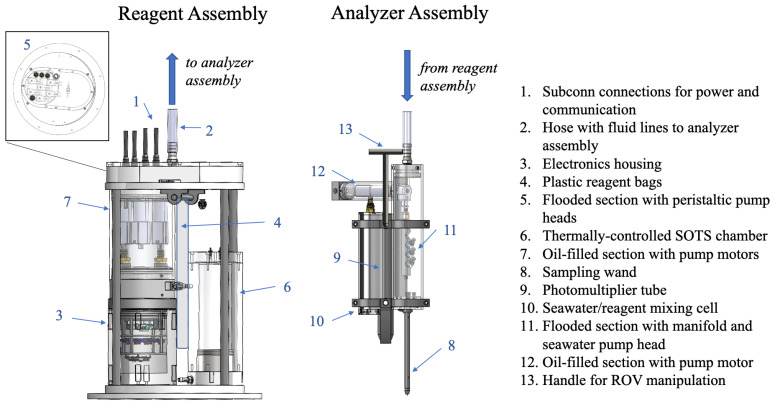
A schematic of the SOLARIS showing labeled parts of the reagent and analyzer assemblies.

**Figure 2:**
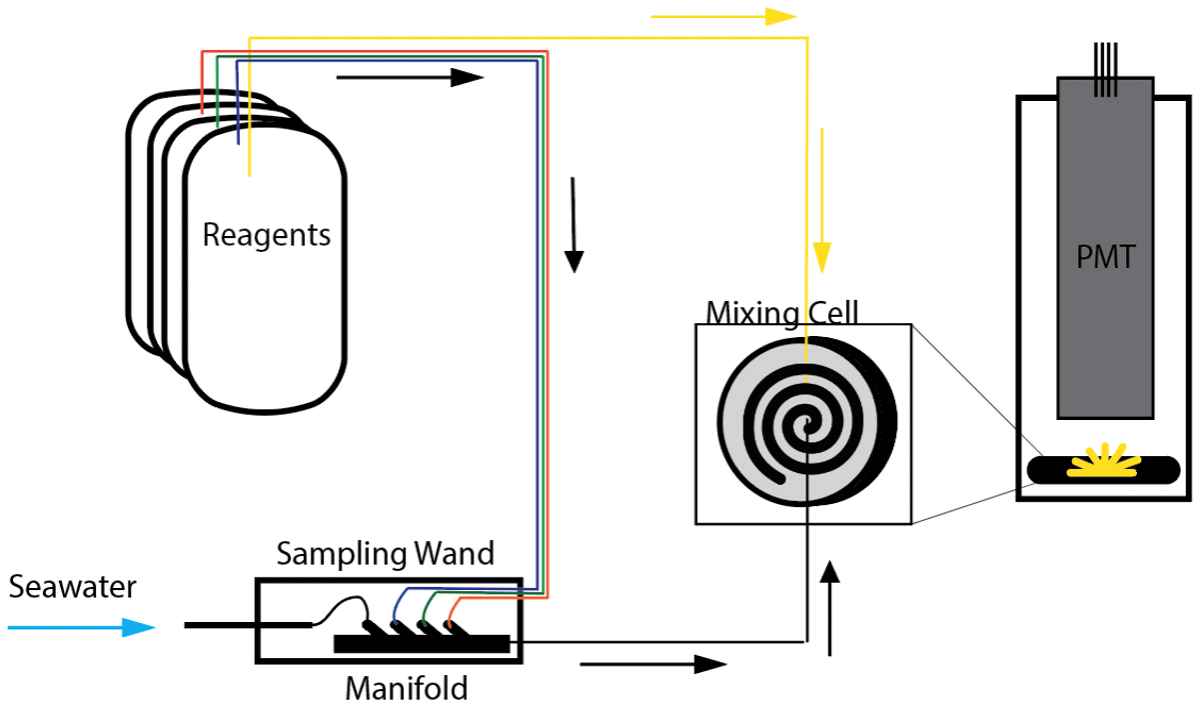
An overview of the pump-driven mixing of fluids in the SOLARIS. Reagents within the reagent assembly, with the exception of MCLA (shown in the yellow line) are directed, and mix with seawater in a manifold in the sampling wand. This flow is then directed to a spiral flow cell where it mixes with MCLA, and is monitored by a photomultiplier tube.

The design of SOLARIS allows for its integration into either rosette or submersible vehicle platforms. SOLARIS is comprised of a main body containing reagents (the reagent assembly) connected to a sampling wand (the analyzer assembly) which continuously draws in seawater for analysis (as seen in Figure 1). Utilizing a sampling wand allows for directed measurements at specific sites of interest, for instance using a manipulator on remotely operated or human-occupied vehicles. Sample $O_{2}^{\cdot -}$ is detected by measuring the chemiluminescence generated upon the reaction between MCLA reagent and superoxide in the respective sample fluid [19]. Reagents are pumped through tubing by peristaltic pumps from the reagent assembly into a spiral mixing cell (as illustrated in Figure 2) in the analyzer assembly, and the chemiluminescent signal is monitored by a photon counting photomultiplier tube (PMT). The instrument is controlled and data is viewed and recorded in real-time using a custom graphic user interface.

#### 2.1.1. Mechanical & Fluidics

The rapid nature of superoxide decay necessitates close proximity of the analysis to the sampling location, a requirement that drove many aspects of the mechanical design of the instrument. SOLARIS is comprised of two assemblies: a larger main reagent assembly and a smaller nimbler analyzing assembly which are connected together via tubing and electrical lines of variable length. Separation of these two assemblies makes it easier to precisely target a sample location with the inlet wand, while the bulk of the instrument remains in a fixed location. Components are separated into each of these assemblies based on required fluidic pathways, reagent delivery and chemiluminescent analysis. Each assembly includes an air-filled pressure tolerant housing (tested to 8925 psi, approximately 5950 m depth), an oil-compensated volume, and a flooded section.

On the reagent assembly the oil volume houses six pump stepper motors (Applied Motion, HT11-021-G022). The shafts of the pump motors extend into a flooded section at the top of the reagent assembly, and there, each drive a peristaltic pump head (Williamson Co. (Taylor, TX, USA), Series 100) with Norprene tubing (3 mm ID × 6mm OD). These pumps deliver reagents (described in Table 1.) from sterile plastic bags (Thermo Scientific, Labtainer P/N SH3071403, 0.5 or 2L volumes) within the reagent assembly to the analyzer assembly through polyethylene tubing (1/16’’ ID; 1/8” OD). The air-filled electronics housing on the reagent assembly contains all the electronics (described in more detail below) required to control and collect data from the instrument. Informed by results of both lab and in situ tests, after the first field deployments (described below) of SOLARIS, the plastic reagent bag holding the calibration solution was moved from its position with the other reagent bags to an insulated and thermally-controlled chamber which was added to the reagent assembly. The reagent bag is held inside the chamber where it is kept at 25 °C with fluid lines that connect it to one of the peristaltic pumps. The volume surrounding the reagent bag is a flooded section which is only open to the ambient water through a pinhole. The purpose of the pinhole is to allow water to back-fill the volume surrounding the reagent bag as fluid from the bag is pumped out, while also keeping the inside of the chamber thermally isolated. The chamber temperature is maintained at 25 °C using two heaters located at the base of the chamber, and the temperature is monitored using a thermistor probe.

The main components of the analyzer assembly consist of a flooded-section containing a peristaltic pump head and a fluidic manifold, an oil-filled section for the pump motor, the PMT housing with a mixing cell, and a sampling wand. At the analyzer assembly, fluids pumped from the reagent assembly are combined in the manifold with sample seawater which is pumped in through a filter tip (0.025 cm holes) located at the end of the sampling wand. All reagent lines that enter the manifold are connected by inert check valves that prevent back-flow and unintended mixing. The output of the reagent manifold is routed directly to the mixing cell being monitored by the photomultiplier tube for changes in light intensity.

Altogether, the analyzer assembly serves as a movable sampling wand with a handle that can be positioned by an ROV manipulator or fixed in a desired position. The reagent and analyzing assemblies are connected together by a flooded length of 1-inch OD Tygon tubing that contains and protects the fluid lines that travel between the assemblies, as well as electrical cables that connect the reagent assembly electronics to the PMT housing and the seawater pump. All fluid connections are made using acetal or peek barbed or flangeless ¼-28 fittings (IDEX).

#### 2.1.2. Electronics and Software

The electronics for SOLARIS are centered around a custom PCB containing a microcontroller (Microchip ATSAMD51) and power distribution electronics. The microcontroller handles communication between a user PC and various components including seven pump motor drivers and the PMT. It also records data on an onboard SD card, and it can store routines that allow the system to operate autonomously if direct user control is not possible. Main instrument power is supplied with external 24 VDC through a four-pin bulkhead (Subconn MCBH4M) on the reagent assembly, and it is regulated or directly distributed to seven motor drivers (Allmotion, P/N: EZ10EN), a temperature controller for the isothermal reagent chamber (Wavelength Electronics, RHM5K-CH), the microcontroller, and the PMT. Communication from the user computer and the microcontroller is handled via RS232 through an eight-pin bulkhead (Subconn MCBH8M) also on the reagent assembly. The microcontroller then communicates to the motor controllers via RS485 and PMT via RS232.

A graphical user interface was designed in Matlab to communicate with the instrument, allowing use of a PC or tablet computer to operate individual fluid pumps, as well as vary individual flow rates. The interface also plots the PMT signal in real-time, and allows the user to annotate sampling events at specific points in time. The user also has the option to upload autonomous routines defined by sequential pump states, flow rates, and durations that will run automatically when the instrument is powered on.

### 2.2. Method of Analysis

SOLARIS measures $O_{2}^{\cdot -}$ via the enhanced chemiluminescence generated upon reaction with the probe methyl Cypridina luciferin analogue (MCLA). This method has frequently been used to detect ROS because MCLA is highly specific and sensitive to its reaction with $O_{2}^{\cdot -}$[19]. The chemiluminescent signal generated upon the reaction between $O_{2}^{\cdot -}$ and MCLA is detected by photomultiplication. The raw photon count is then converted to a $O_{2}^{\cdot -}$ concentration by calibration with a standard solution of SOTS-1 [9] (see Table 1).

### 2.3. Operations

There are two principal modes in which the SOLARIS operates: calibration and seawater analysis. Within the timespan of a deployment, calibrations are run periodically, briefly interrupting analysis of environmental seawater.

#### 2.3.1. Calibration Approach

SOLARIS was designed to perform in situ calibrations, which are necessary given the long deployment (up to 8 h) period, and gradient of environmental conditions (light, temperature, etc.) encountered during a single deployment. To generate a calibration factor (photon count/concentration of $O_{2}^{\cdot -}$) a solution of SOTS-1 (see Table 1) which has a known superoxide concentration, is routinely measured.

The use of SOTS-1 as a time-dependent $O_{2}^{\cdot -}$ source was adapted from previously outlined methods [9]. The concentration of superoxide in a SOTS-1 solution at any given time depends on the initial concentration of SOTS-1 and the rate of decay of superoxide both by first-order reactions with metals, and via uncatalyzed dismutation. Here we used standard solutions of 100 µM SOTS-1 prepared in reagent background seawater that is amended with DTPA (75 µM) to sequester trace metals, thereby eliminating the need to take first-order reaction rates into account. Thus, the rate equation for the formation of $O_{2}^{\cdot -}$ from SOTS-1 has the solution [9]:

(1) $${\left[O_{2}^{\cdot -}\right]}_{i}=\sqrt{\frac{0.4k{\left[\mathrm{SOTS}\right]}_{0}\;e^{-kt}}{2k_{D}}}$$

**Figure 3:**
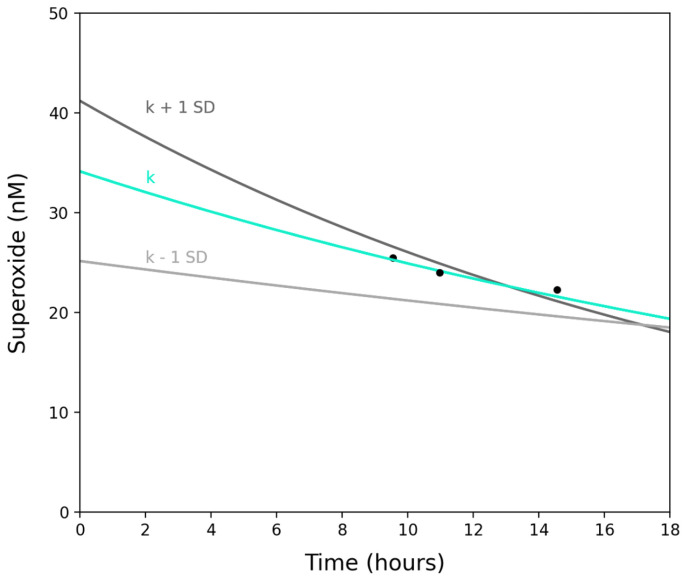
A model of the instantaneous $O_{2}^{\cdot -}$ concentration produced by a diluted 100 µM SOTS-1 solution as a function of time. The turquoise line gives the evolution of $O_{2}^{\cdot -}$ produced by the thermal degradation of SOTS-1 for a decay constant at 10°C. The gray lines depict the impact of the uncertainty (±1 SD) in the degradation constant. The black points show $O_{2}^{\cdot -}$ concentrations estimated from independent KO${}_{2}$ calibrations.

The concentration of $O_{2}^{\cdot -}$ in the standard solution is modeled in Figure 3 for an uncatalyzed second-order dismutation rate constant, *k_D_*, of 3.0 × 10${}^{5}$ M${}^{-1}$ s${}^{-1}$, and temperature-dependent decay constant, *k*, for SOTS-1 of 1.7 ± 0.8 × 10${}^{-5}$ [9]. These constants were chosen as the most relevant to the temperature of the environment being sampled.

During calibration, a background signal is first generated by mixing MCLA (6.3 mL/min) with reagent background seawater (6.3 mL/min). Once a steady (visually identified by user in real-time as a flat line with minimal fluctuations) signal has been obtained, the SOTS-1 pump (1.5 mL/min) is activated, and mixes in with the reagent background seawater (4.8 mL/min) (at a ratio of approximately 1:3.2) and MCLA (6.3 mL/min) in the spiral flow cell. After the elevated signal has been collected for 20 s, SOD is added (1.5 mL/min), drawing the signal down below baseline, completing the calibration sequence. With knowledge of the time-dependent $O_{2}^{\cdot -}$ concentration in the SOTS-1 solution, and the in-situ PMT signal, a photon count per $O_{2}^{\cdot -}$ concentration calibration factor can be obtained. The frequency of calibrations is left to the users discretion, however, given the temperature effects of MCLA (described in Section 3.2) it is advised that they are done whenever shifts in temperature are greater than 4 °C, or changes in the seawater chemistry (e.g., metal concentrations) are encountered.

The accuracy of the model estimated concentrations of $O_{2}^{\cdot -}$ in the standard solution was independently evaluated in lab by calibrations using a KO${}_{2}$ method [18,20]. As in previous studies that used MCLA generated chemiluminescence for $O_{2}^{\cdot -}$ quantification, KO${}_{2}$ standard solutions were created for each calibration by dissolving KO${}_{2}$ in a basic solution (0.3N NaOH, 75 µM DTPA, pH = 12.5).

**Figure 4:**
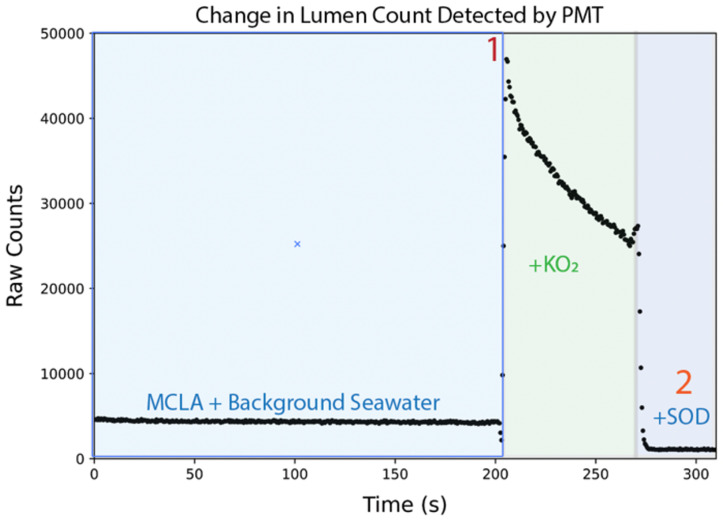
KO${}_{2}$ calibrations were done to test the predicted $O_{2}^{\cdot -}$ concentration generated by the thermal degradation of SOTS-1. A KO${}_{2}$ calibration begins with the collection of a background signal. With the difference in absorbance at 240 nm of a KO${}_{2}$ solution before (1) and after the addition of SOD (2), and the observed slope of the $O_{2}^{\cdot -}$ decay, a calibration factor (raw photon count/ nM $O_{2}^{\cdot -}$) can be estimated.

The KO${}_{2}$ calibration (depicted in Figure 4) procedure begins with the generation of a background signal in filtered and DTPA-amended seawater, which establishes the luminescence in the absence of $O_{2}^{\cdot -}$. Background seawater solution is drawn directly through the sample wand from a centrifuge tube and the signal is recorded.

When 20 mL of background seawater remain in the tube a 4 uL aliquot of KO${}_{2}$ stock solution is added and briefly mixed. A contemporaneous measurement of the absorbance of the KO${}_{2}$ stock in a quartz cuvette at 240 nm is made on a UV-Vis spectrophotometer as the resulting photon peak is recorded. The signal of the $O_{2}^{\cdot -}$ decay in the KO${}_{2}$-spiked solution is measured for 50 to 100 s. When 10 mL of KO${}_{2}$-spiked solution remains in the centrifuge tube an aliquot of SOD solution (4 kU mL${}^{-1}$) is added. At the same time, SOD is added to the quartz cuvette and the change in absorbance is recorded.

The absorbances are converted to molar units using the molar absorption coefficient of $O_{2}^{\cdot -}$ (2183 mol${}^{-1}$ cm${}^{-1}$, pH = 12.5), and used to estimate a calibration factor (photons per concentration of $O_{2}^{\cdot -}$). The raw photon count generated by the reaction between the SOTS-1 solution and MCLA is observed and then converted to a $O_{2}^{\cdot -}$ concentration at several time points. As shown in Figure 3, the resulting concentrations are in good agreement with the model predicted values.

#### 2.3.2. Environmental Analyses: SOLARIS Deployment on CTD Casts

**Figure 5:**
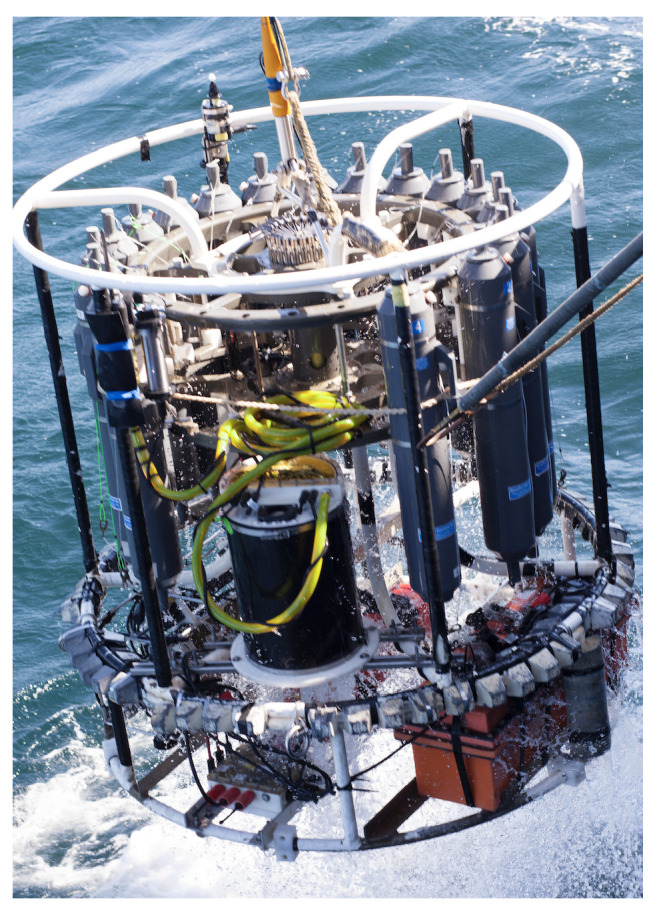
The SOLARIS mounted on a CTD frame for measurements of water column $O_{2}^{\cdot -}$.

During initial field deployments, SOLARIS was mounted on a CTD rosette aboard the R/V Atlantis (see Figure 5). In this configuration power was supplied to the instrument by an external 24VDC (40 amp/h) battery (DeepSea Power & Light, P/N: SB-24/40) mounted to the lower frame of the rosette. The sampling wand was mounted on the inside of the outer rosette frame with the end of the wand pointed upwards. During the cast, SOLARIS was remotely controlled using a custom software interface, which allowed the user to modify pump speeds, and periodically perform calibration routines. In the deployments of SOLARIS shown here, calibrations were conducted at the surface, at the onset of the chemocline, approximately every 3 °C shift in temperature throughout the chemocline, and then twice at depths below to capture changes in temperature and seawater chemistry. Communications with the instrument down the cable involved use of a custom underwater digital modem for conversion of the Ethernet signal for transmission up a single conductor on the CTD cable [21]. During the downcast MCLA (6.3 mL/min) and sample seawater (6.3 mL/min) were continuously mixed into flow cell and luminescence was quantified by the PMT. On the upcast, SOD (see Table 1) was continuously added allowing for baseline correction. Temperature measurements made during CTD casts informed the choice of the temperature dependent degradation constants used to model the concentrations of $O_{2}^{\cdot -}$ in calibration solutions.

## 3. Results and Discussion

### 3.1. Linearity of Response to $O_{2}^{\cdot -}$ Concentration

**Figure 6:**
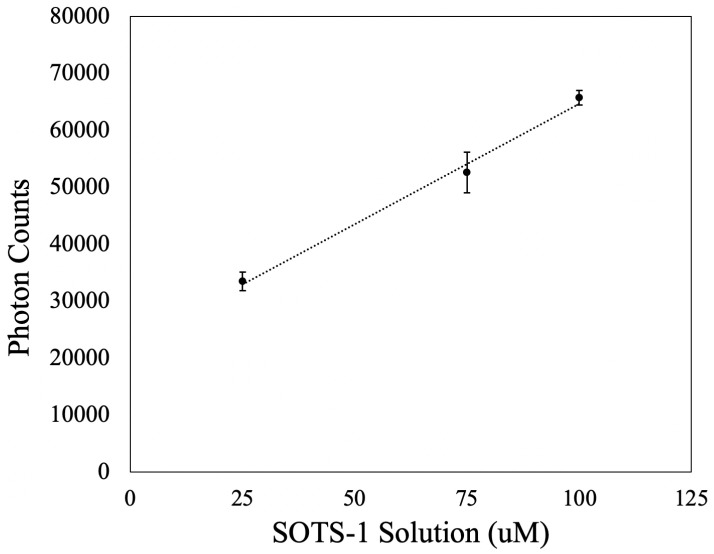
There is a linear relationship between the raw photon count and the concentration of the SOTS-1 solution. The error bars highlight the range in photon counts observed across the duplicate samples.

The linearity in the response of the PMT was tested by sequentially measuring different calibration solutions. Three calibration solutions were prepared in duplicate at 25, 75, and 100 µM SOTS-1 concentration. Each solution was prepared in 50 mL falcon tubes, and stored in the dark (at 21 °C). As seen in Figure 6, there is a linear response (R${}^{2}$ = 0.9933) in the photon counts resulting from the chemiluminescent reaction between MCLA and solutions of increasing SOTS-1 concentration. This indicates that as the SOTS-1 in the initial calibration media decays with time, the temperature-dependent decrease in instantaneous $O_{2}^{\cdot -}$ concentration scales linearly with the photons detected.

### 3.2. Temperature Effects

SOTS-1.The decomposition of SOTS-1 follows a first order temperature-dependent decay. The thermal degradation rate constant (k) has been experimentally determined in previous work [9]. In the field work herein, the temperature during CTD casts ranged from 15 °C at the surface to 4.9 °C at 3000 m. To capture this variability, we have used an average decay constant of 1.7 ± 0.8 × 10${}^{-5}$, which is valid for a temperature range of 10–20 °C [9]. Rate constants below 10 °C have not been reported. The addition of the temperature regulated SOTS chamber implemented after the completion of this initial deployment serves to maintain the SOTS calibration solution at a constant temperature during measurement, thereby mitigating the need for temperature correction.

**Figure 7:**
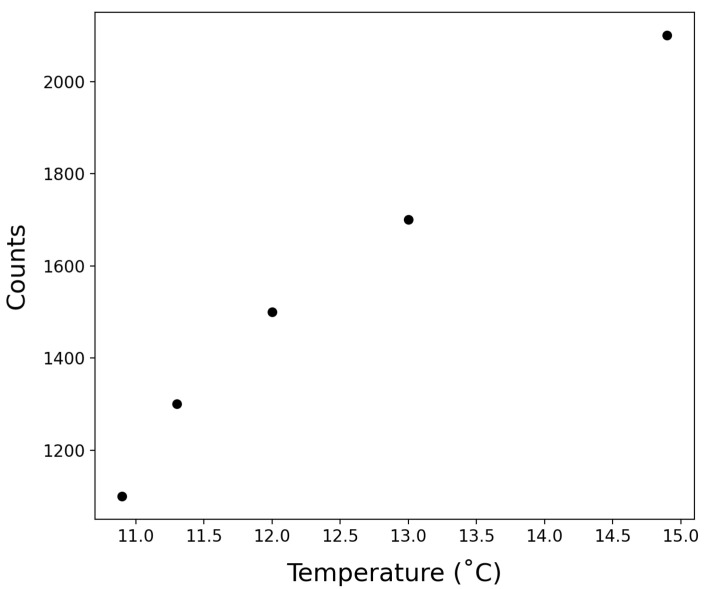
The raw photon counts observed by the PMT from MCLA at different temperatures.

MCLA. As previously recognized [16], there is an increase in the baseline chemiluminescence with temperature (Figure 7). However, since calibrations are done in situ, the calibration factor takes the temperature of the MCLA into account, and is specific to that time of measurement. As such, no additional constraint of this temperature effect was required.

### 3.3. Flow Rate Tests

Pump speeds controlling the flow from the reagent bags and sample wand were independently tested against the speeds that were set in the user interface. Pump speeds operated at room temperature were reproducible over several weeks, and were serviced when drift (for example a difference between set and measured flow speed of approximately 5% or >0.2 mL/min) was observed. Lab experiments carried out in a temperature controlled-room set to 5°C revealed a drop in the measured pump speeds at colder temperatures relative to the set pump speeds. This general trend was characteristic of all pumps, but expressed itself to different degrees, ranging from decreases between 4 and 13% of the set speed. We expect that this drift is likely to occur as temperatures drop throughout the water column, which we speculate could be due to stiffening of the (Viton) tubing at lower temperatures. However, since calibrations are subject to the same shifts in pump speed as in-situ measurements, we also expect this variability to be captured by our calibration factor.

**Figure 8:**
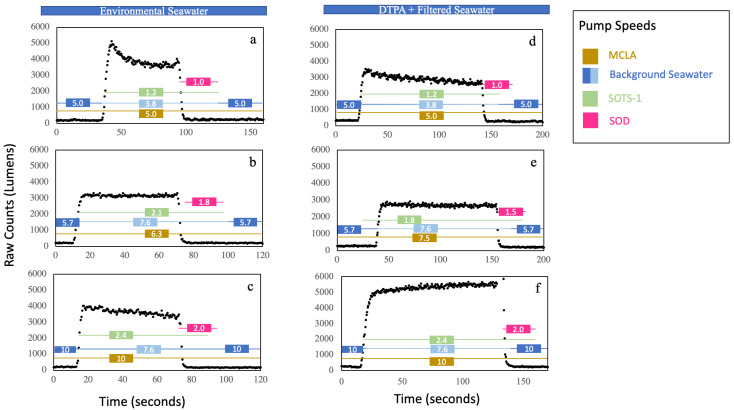
A series of calibrations done to test how the photon count detected by the PMT varied in response to different standard concentrations, flow speeds (given in the rectangular box for each respective reagent), and mediums for standard dilution (environmental seawater or DTPA amended and filtered seawater). Calibration solutions were diluted in-line with environmental seawater in (**a**–**c**), and with DTPA amended and filtered seawater in (**d**–**f**). The concentration of SOTS was decreased in tests (**b**,**e**) in comparison with (**a**,**c**). The effect of background medium flow speed was tested in (**e**,**f**). In (**a**) the initial decrease in signal is an effect of the lines not being filled with fluid when the test began, this can be avoided by priming the line in advance.

A series of tests were done to examine the effect of flow speed on raw photon counts recorded by the PMT (Figure 8). Calibrations did not significantly differ when dilution of the SOTS-1 solution occurred from filtered + DTPA amended BGSW versus environmental seawater drawn through the sample wand. The time lag between the initiation of the SOTS pump and the observed response in signal was tested at various pump speed configurations. For field work, continuous measurements were made by running MCLA and sample at 6.3 mL/min which resulted in a change in signal 20-25 s after the SOTS-1 pump had been started. Adding SOD into the mixing line returned the signal to baseline, which exhibited no dependence on the flow speed (Figure 8).

### 3.4. $O_{2}^{\cdot -}$ Depth Column Measurements

**Figure 9:**
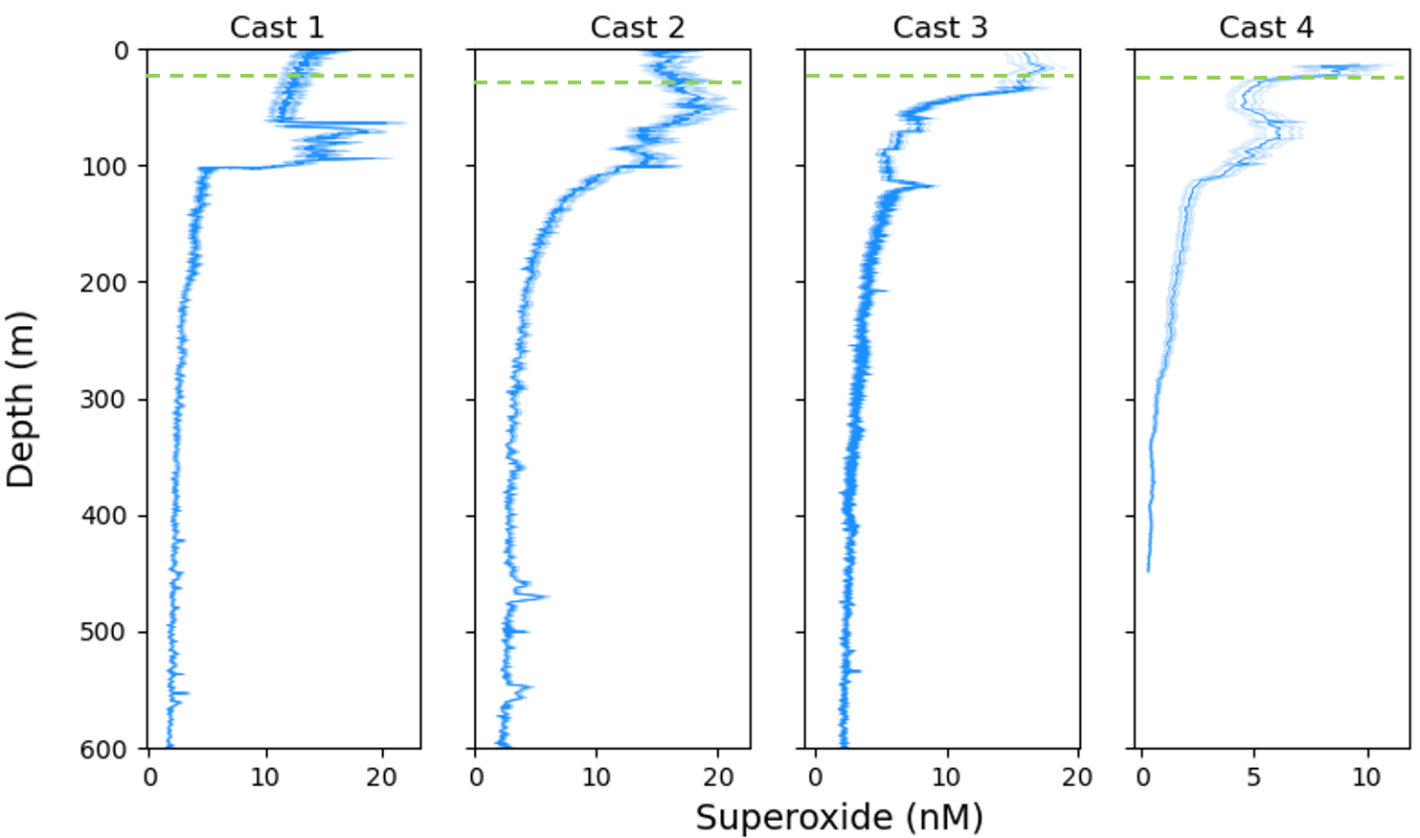
$O_{2}^{\cdot -}$ concentrations (nM) as a function of depth collected with the SOLARIS during four different CTD casts off of western California. The light blue lines highlight the uncertainty range based on a Monte Carlo simulation (±1 S.D) of all calibration factors obtained along the cast. The green dashed line depicts the depth of the surface mixed layer.

SOLARIS was used to capture the first in situ depth profiles of $O_{2}^{\cdot -}$ off of western California in October 2019 (Figure 9). Seawater was analyzed continuously on the downcast, with calibrations conducted at specific depth intervals. During the upcast the same configurations were used as on the downcast, with the addition of SOD into the mixing manifold. The average photon count collected on the upcast with the addition of SOD was subtracted from the raw photon counts of the MCLA and seawater signal to remove the background.

The profiles show that $O_{2}^{\cdot -}$ persists throughout the water column with the highest concentrations (between 15 and 25 nM) found within the first 80 m of the surface. Previous measurements of surface ocean superoxide, range from below detection limit to approximately 1.8 nM [16,17,19,22]. Due to a lack of in situ instrumentation, previous estimates of superoxide have been based off of water collected from a CTD rosette, or extrapolated from decay rate measurements in filtered water. Since the measurements made herein were done in situ with only coarsely filtered (0.025 cm) waters from the sampling wand, the signals represent a combination of light-dependent and light-independent reactions associated with biotic and abiotic processes in the dissolved and particulate phase.

The elevated levels observed in the surface waters reflect the importance of light-dependent pathways, likely a combination of abiotic (e.g., photooxidation of DOM) and biotic (e.g., photosynthetic microbes) production. The range in surface concentrations illustrates the variations in chemical and biological processes in the different sampling locations, but may also be in part a function of the sampling time and related light intensity. All profiles show a decline in $O_{2}^{\cdot -}$ concentrations below the euphotic zone, and a stabilization at low but non-zero concentrations below 200 meters.

**Figure 10:**
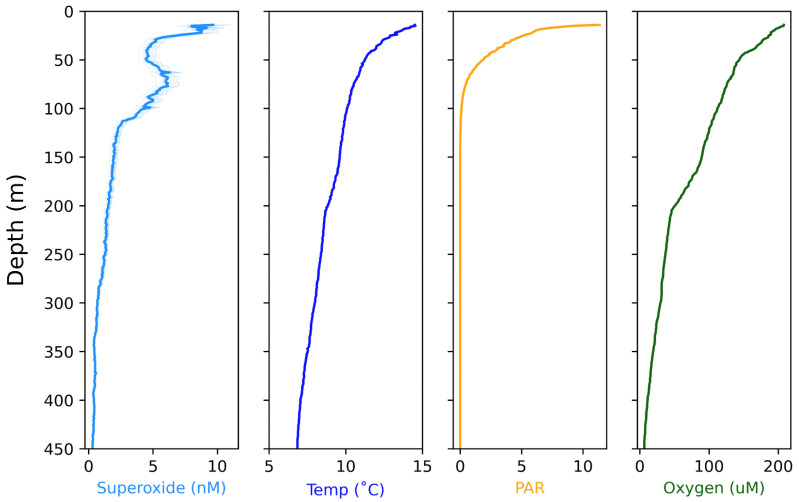
The $O_{2}^{\cdot -}$ concentrations with depth for Cast 4, in comparison with temperature, PAR, and oxygen data from the CTD.

Interestingly, the dynamics of our measured $O_{2}^{\cdot -}$ profiles do not closely correlate with the patterns in temperature, oxygen, or incident light data collected during the contemporaneous CTD cast (Figure 10). We observed secondary peaks in $O_{2}^{\cdot -}$ concentrations during casts 1, 2, and 4 at depths below the photic zone, suggesting that photochemical and phototrophic reactions are not the dominant production processes at these depths (Figure 9). An alternative explanation for the source of $O_{2}^{\cdot -}$ could be particle-associated production, which has previously been observed in various field studies [16,19,22,23,24]. Extracellular $O_{2}^{\cdot -}$ generation by heterotrophic bacteria and at mineral surfaces proceeds in the absence of light [25,26,27,28,29], and thus at a depth of around 70 m elevated superoxide could arise as a result of high particle flux. Yet, the relative contribution of various (a)biological processes responsible for the superoxide profiles warrants future targeted exploration.

## 4. Conclusions

Here, we have developed a versatile chemiluminescent sensor, capable of continuous data collection at depth. While it has been outfitted to measure superoxide, its customizable nature makes it suitable to carry out any number of chemiluminescent measurements (e.g., hydrogen peroxide or Fe(II)). Our results highlight the ability of SOLARIS to make measurements of $O_{2}^{\cdot -}$ throughout the ocean water column, as well as capture small-scale variations associated with biological activity. The power of this high-resolution capability promises to enable unprecedented examination of $O_{2}^{\cdot -}$ across a variety of marine ecosystems and will help to decipher variations in superoxide production between different species of deep-sea corals, sponges, and other organisms. The length of time that SOLARIS can be deployed is currently limited by the volume of reagents held in the bags (MCLA, SOD), and the constraints of the CTD or ROV/HOV platform (6 to 8 h for Alvin) from which SOLARIS is operated. At this point, we have not tested SOLARIS for extended deployments, but future exploration of its temporal limitations is needed. The advent of continuous $O_{2}^{\cdot -}$ data at depth holds the potential for a better understanding of its production and roles in the marine environment.

## Figures and Tables

**Table 1:** The four reagents used for the analysis of superoxide in seawater.

Reagent	Purpose	Description
MCLA	A chemical probe which produces a chemiluminescent signal upon reaction with $O_{2}^{\cdot -}$	MCLA reagent (4 µM) is prepared in a sodium acetate buffer to a pH of 6 and amended with diethyleneaminepentaacetic acid (DTPA) (50 µM)
Background Seawater	Baseline signal for calibrations.	500 mL of filtered seawater is collected at the site of measurement and amended with DTPA (75 µM) for 12 h.
Superoxide Thermal Source (SOTS-1)	An azo compound that predictably decomposes to produce $O_{2}^{\cdot -}$ at a rate defined by temperature. Used as a superoxide standard.	A 100 µM SOTS-1 solution is prepared by dissolving 5 mg SOTS-1 in 500 μL of dimethyl sulfoxide (DMSO), and diluted to 150 mL with DI.
Superoxide dismutase (SOD)	Used to confirm presence of $O_{2}^{\cdot -}$	SOD is an enzyme responsible for the degradation of $O_{2}^{\cdot -}$ into hydrogen peroxide and molecular oxygen. SOD solutions are prepared by adding 4 kU mL${}^{-1}$ in deionized water.

## Data Availability

All shipboard data is openly available in R2R at Cruise ID AT 42-18 (Cruise DOI:10.7284/908575). The superoxide and testing data in this study is available in the attached Supplementary File.

## Supplementary Materials

The following are available online at https://www.mdpi.com/article/10.3390/s22051709/s1.
